# DriveLLaVA: Human-Level Behavior Decisions via Vision Language Model

**DOI:** 10.3390/s24134113

**Published:** 2024-06-25

**Authors:** Rui Zhao, Qirui Yuan, Jinyu Li, Yuze Fan, Yun Li, Fei Gao

**Affiliations:** 1 College of Automotive Engineering, Jilin University, Changchun 130025, China; rzhao@jlu.edu.cn (R.Z.); yuanqr23@mails.jlu.edu.cn (Q.Y.); lijy1522@mails.jlu.edu.cn (J.L.); fanyz23@mails.jlu.edu.cn (Y.F.); 2 Graduate School of Information and Science Technology, The University of Tokyo, Tokyo 113-8654, Japan; li-yun@g.ecc.u-tokyo.ac.jp; 3 National Key Laboratory of Automotive Chassis Integration and Bionics, Jilin University, Changchun 130025, China

**Keywords:** autonomous driving, behavior decision, visual language model, instruction fine-tuning

## Abstract

Human-level driving is the ultimate goal of autonomous driving. As the top-level decision-making aspect of autonomous driving, behavior decision establishes short-term driving behavior strategies by evaluating road structures, adhering to traffic rules, and analyzing the intentions of other traffic participants. Existing behavior decisions are primarily implemented based on rule-based methods, exhibiting insufficient generalization capabilities when faced with new and unseen driving scenarios. In this paper, we propose a novel behavior decision method that leverages the inherent generalization and commonsense reasoning abilities of visual language models (VLMs) to learn and simulate the behavior decision process in human driving. We constructed a novel instruction-following dataset containing a large number of image–text instructions paired with corresponding driving behavior labels, to support the learning of the Drive Large Language and Vision Assistant (DriveLLaVA) and enhance the transparency and interpretability of the entire decision process. DriveLLaVA is fine-tuned on this dataset using the Low-Rank Adaptation (LoRA) approach, which efficiently optimizes the model parameter count and significantly reduces training costs. We conducted extensive experiments on a large-scale instruction-following dataset, and compared with state-of-the-art methods, DriveLLaVA demonstrated excellent behavior decision performance. DriveLLaVA is capable of handling various complex driving scenarios, showing strong robustness and generalization abilities.

## 1. Introduction

In recent years, autonomous driving has made significant progress and has rapidly become one of the most promising fields in modern technology, with the potential to transform global transportation systems [1,2]. The ultimate goal of autonomous driving is to achieve human-level driving. As a core challenge of autonomous driving systems, behavior decision-making focuses on devising behavior strategies for autonomous vehicles that are consistent with human driving processes, such as lane change and speed adjustment, to ensure that they can intelligently respond to complex and dynamic traffic environments.

The challenge of behavior decisions lies in the need to adapt to diverse driving scenarios and make reasonable driving strategies. In traditional autonomous driving systems, behavior decision-making is typically based on rule-based methods [3,4,5,6], which determine driving strategies through pre-designed explicit rules to handle various scenarios. These methods have a high degree of interpretability but may fail when encountering scenarios not covered by the rules or previously unseen extreme driving scenarios, especially long-tail edge cases [7,8] (i.e., irregular behavior of road users, atypical obstacles, and unusual environments).

To ensure that vehicles can handle a wide range of driving scenarios, learning-based methods [9,10,11,12,13,14,15,16] have become the mainstream approach in modern autonomous driving systems for behavior decisions. These methods are data-driven, using human driving data to train decision models. Despite demonstrating excellent performance, their operation resembles a “black box”, meaning the entire decision process lacks intuitive interpretability and is difficult for humans to understand, leading to widespread public distrust and legal concerns. Moreover, current rule-based and learning-based methods lack a certain degree of social intelligence [17,18,19,20]. They treat autonomous driving as a mechanical task, viewing the interaction between the vehicle and its surroundings as simple kinematic cooperation [21,22], while neglecting the social cognitive context that is crucial for understanding driving behavior.

The rapid development of Large Language Models (LLMs) brings hope for addressing these challenges. LLMs possess powerful generalization and commonsense reasoning abilities, enabling them to infer information from previously unseen scenarios. This potential can help autonomous vehicles handle long-tail edge cases. Since LLMs are pretrained on vast amounts of interdisciplinary data, including understanding social interactions and human behavior, they may bridge the social intelligence gap present in autonomous driving systems [23]. Furthermore, to achieve an understanding of visual information, Vision Language Models (VLMs) build on LLMs by mapping multimodal inputs from images, videos, and other spatial data into the text domain, enabling VLMs to process and comprehend these multimodal data as text.

This paper aims to employ VLMs for behavior decision tasks in autonomous driving. We reference human driving paradigms to identify three essential capabilities for VLMs: (1) **Observation**: The model can accurately perceive the surrounding environment and changes in specific driving scenarios. (2) **Reasoning**: Based on observational information, the model can use common sense and experience to reason and make decisions. (3) **Explanation**: The model’s reasoning and decision process can be clearly explained.

Based on these three characteristics, this paper proposes a novel Vision Language Model, DriveLLaVA, to learn and simulate the behavior decision process in human driving. DriveLLaVA uses multimodal perception information from the ego vehicle as prompt input to accurately plan future driving behavior. To ensure the consistency of DriveLLaVA’s reasoning outputs with human behavior decisions, we constructed a highly interpretable instruction-following dataset based on the nuScenes [24] dataset. This dataset contains a large number of image–text instructions paired with corresponding driving behavior labels for fine-tuning the model.

The main contributions of this paper are as follows:

(1) We construct an instruction-following dataset containing a large number of interpretable image–text instructions and driving behavior labels. The image–text instructions provide multimodal perception information, including surrounding traffic participants’ observation information, ego vehicle motion information, and environmental visual information. The driving behavior labels detail the ego vehicle’s actual future driving behaviors and are represented as text-based meta-action sets. This novel dataset comprehensively reflects the behavior decision process in human driving.
(2) We propose a novel Vision Language Model, DriveLLaVA, specifically for autonomous driving behavior decision tasks and fine-tuned on the constructed instruction-following dataset. During fine-tuning, adapter technology is employed to efficiently optimize model parameters, significantly reducing training costs. The proposed VLM can efficiently infer driving behavior strategies consistently with human behavior.
(3) We evaluate this method on multiple experiments, and DriveLLaVA outperforms all baseline methods, demonstrating excellent behavior decision performance. Additionally, DriveLLaVA can handle various unseen complex driving scenarios through few-shot generalization, showing strong robustness and generalization abilities.

## 2. Related Work

### 2.1. Learning-Based Autonomous Driving Behavior Decision

As the top-level planning aspect of autonomous driving, behavior decision-making establishes short-term driving strategies by evaluating road structures, adhering to traffic rules, and interpreting the activities of other traffic participants. The primary output of the behavior decision task is a limited set of actions suitable for driving scenarios [25], including basic driving behavior strategies (such as car-following and lane-changing).

Currently, learning-based autonomous driving behavior decision-making mainly includes Imitation Learning (IL) methods and Deep Reinforcement Learning (DRL) methods. IL methods, which learn from expert demonstrations to replicate human-like driving behavior, have become a prominent technology in the field of autonomous driving behavior decision. Tian et al. [9] proposed a personalized end-to-end IL decision method that efficiently learns a Model Prediction Control (MPC)-based driver-specific lane-changing policy from a few driver demonstrations. Ozcelik et al. [10] employed Generative Adversarial Imitation Learning (GAIL) and Curriculum Learning (CL) to mimic expert behavior in highway scenarios, aiming to achieve driving behavior similar to human drivers. Bhattacharyya et al. [11] described the use of GAIL and its extensions, such as Parameter Sharing-GAIL (PS-GAIL), Reward-Augmented Imitation Learning (RAIL), and Burn-InfoGAIL, to simulate highway driving behavior, replicate human demonstrations and generate realistic, emergent behavior in traffic flows.

Furthermore, researchers have explored the application of DRL methods in autonomous driving behavior decisions, with DRL integration fostering a series of advances across various driving scenarios. Kamran et al. [12] proposed an efficient DRL-based decision process to provide high-level strategies and specify the operation mode of low-level planners in merging scenarios. Valiente et al. [13] extended the use of Deep Q-Network (DQN) to facilitate autonomous behavior navigation in diverse scenarios such as highways and roundabouts. Zhang et al. [14] developed a two-stage lane-changing behavior decision system, demonstrating a complex balance between rule-based and DRL methods, enhancing cooperation between vehicles and improving overall safety and efficiency in traffic scenarios. Toghid et al. [15] introduced Social Value Orientation (SVO) into the DRL paradigm, enabling AVs to form alliances, guide traffic, and actively influence the behavior of Human-driven Vehicles (HVs) in mixed autonomy environments. Wang et al. [16] improved and stabilized Adversarial Inverse Reinforcement Learning (AIRL) by adding semantic rewards, making it suitable for challenging behavior decision tasks in highly interactive autonomous driving environments. However, current learning-based methods also have inherent limitations. On the one hand, the entire behavior decision process resembles a “black box”, lacking intuitive interpretability and transparency. On the other hand, due to the neglect of the social cognitive context in the driving process, there is a gap in social intelligence.

This paper aims to employ VLM for autonomous driving behavior decision tasks using multimodal perception information from ego vehicles as input to accurately plan future high-level driving behavior. To enhance the interpretability of the entire decision process, this work designs future driving behavior as a text-based meta-action set, with each meta-action subdivided into a combination of direction and speed components. Additionally, VLM is pretrained on vast amounts of interdisciplinary data, possesses common knowledge of the human world, and can address the social intelligence gap present in current methods.

### 2.2. LLM/VLM in Autonomous Driving

Currently, LLMs have achieved rapid development, showing significant potential in simulating human intelligence. These models are trained on vast amounts of internet data to understand and generate human-like text, demonstrating outstanding performance in natural language processing. The most notable features of LLM are their emergent capabilities, such as In-Context Learning (ICL) [26], Instruction-Following [27], and chain-of-thought (CoT) reasoning [28]. GPT [26] was a pioneering work that proposed using the Generative Pretrained Transformer (GPT) to address text understanding and generation problems. Subsequent versions, GPT-3.5 and GPT-4 [29], have also demonstrated impressive conversational and reasoning abilities. Recently released LLMs, such as PaLM [30], Vicuna [31], LLaMA and LLaMA 2 [32,33], generate corresponding text feedback based on human-followed instructions to better leverage LLM’s instruction-following capabilities. Furthermore, to handle various input types beyond text, research on VLM has also gained extensive academic attention. These models combine LLM with visual encoders, enabling them to efficiently perform various tasks involving images, videos and audio data. Based on the strong weights of natural language models, some recently proposed VLM, such as CLIP [34], PaLM-E [35], VisualBERT [36], simVLM [37], and Flamingo [38], possess excellent cross-modal understanding capabilities by aligning on large-scale multimodal datasets. Typically, VLMs use Q-former or linear mapping to align image features with the embedding space of language models. Blip-2 [39] uses Q-former to project multimodal inputs into the text space, while LLaVA [40] and Qwen-VL [41] train a fully connected layer as a projector to align image features with text features.

In the field of autonomous driving, LLM/VLM have the potential to understand traffic scenarios, improve the driving decision process, and revolutionize interactions between humans and vehicles. Trained on large amounts of autonomous driving data, they can extract valuable information from various sources such as maps, text annotations, and traffic rules to enhance the navigation and planning of autonomous vehicles, adapting to ever-changing traffic scenarios. Fu et al. [42] used GPT-3.5 for explanation and interaction in simple simulated scenarios, initially validating the applicability of LLM in autonomous driving tasks. Mao et al. [43] redefined motion planning as a language modeling problem, utilizing GPT-3.5 to describe high-precision vehicle trajectory coordinates and internal reasoning processes in natural language. Tian et al. [44] introduced DriveVLM, which integrates a chain-of-thought module combination for autonomous driving scenario description and analysis, achieving hierarchical planning functions. Sima et al. [45] designed DriveLM by fine-tuning VLM on private datasets to predict high-level driving plans (e.g., move straight, turn left).

This paper proposes a novel VLM, DriveLLaVA, to learn and simulate the behavior decision process in real human driving. Leveraging the strong generalization and commonsense reasoning abilities of VLM, DriveLLaVA can handle challenging long-tail driving scenarios. Additionally, DriveLLaVA offers better decision transparency and human-like reasoning compared to current methods, primarily due to our novel instruction-following dataset based on the nuScenes dataset.

## 3. Problem Definition

In the real world, human drivers observe their surroundings and other traffic participants, predicting their future movements while driving a vehicle. Based on these observations and predictions, human drivers make timely judgments, plan driving behaviors, and execute corresponding actions to control the vehicle’s future motion. Inspired by human driving thinking, this paper proposes an innovative method that leverages VLM to learn and simulate the decision process in human driving. This method uses multi-modal perception information from the ego vehicle as prompt input, including surrounding traffic participants’ observation information, ego vehicle motion information, and environmental visual information, guiding the VLM to accurately plan the ego vehicle’s future driving behavior.

Behavior decision-making aims to accurately plan the vehicle’s future high-level driving behaviors, such as lane changes and speed adjustment. To achieve this task, we use surrounding traffic participants’ observation information O, ego vehicle motion information E, and front-view camera images I as inputs to guide the VLM in outputting the future driving behavior of ego vehicle A:

(1) $$A=F_{\mathrm{VLM}}\left(O,E,I\right)$$

where $F_{\mathrm{VLM}}$ represents the decision function. Observation information O includes category name $c^{i}$ of surrounding traffic participants *i*, i∈1,…,N (e.g., vehicles, pedestrians, and animals), location $\left(x_{t_{obs}}^{i},y_{t_{obs}}^{i}\right)$ at the current observation time $t_{obs}$, and predicted trajectory for the next $T_{pred}^{\tau }$ timesteps, which is defined as

(2) $$O=\overset{N}{\underset{i=1}{⋃}}\left(c^{i}\cap \left\{\left(x_{t_{obs}}^{i},y_{t_{obs}}^{i}\right),\left(x_{t_{obs}+1}^{i},y_{t_{obs}+1}^{i}\right),\ldots ,\left(x_{t_{obs}+T_{pred}^{\tau }}^{i},y_{t_{obs}+T_{pred}^{\tau }}^{i}\right)\right\}\right)$$

Ego vehicle motion information E is a set containing current states s and historical trajectory h:

(3) E={s,h}

where current states $s=\{x_{t_{obs}}^{e},y_{t_{obs}}^{e},v_{t_{obs}}^{e},a_{t_{obs}}^{e},w_{t_{obs}}^{e},\theta_{t_{obs}}^{e}\}$ cover location $x_{t_{obs}}^{e}$, $y_{t_{obs}}^{e}$, velocity $v_{t_{obs}}^{e}$, acceleration $a_{t_{obs}}^{e}$, yaw rate $w_{t_{obs}}^{e}$ and steering angle $\theta_{t_{obs}}^{e}$ of the ego vehicle at the current observation time $t_{obs}$, and historical trajectory $h=\{\left(x_{t_{obs}-T_{hist}}^{e},y_{t_{obs}-T_{hist}}^{e}\right),\ldots ,\left(x_{t_{obs}-1}^{e},y_{t_{obs}-1}^{e}\right)\}$ records the motion trajectory over the past $T_{hist}$ timesteps. The front-view camera images I provide rich environmental visual information about road facilities, traffic signs, and more.

The future driving behavior A can be expressed as a set of meta-actions for ego vehicle over the future $T_{pred}^{a}$ timesteps:

(4) $$A=\left\{a_{t_{obs}+1},a_{t_{obs}+2},\ldots ,a_{t_{obs}+T_{pred}^{a}}\right\},\mathrm{where}\;a:=a^{dir}\times a^{spd}$$

where meta-action a is defined as a combination of direction judgment $a^{dir}$ and speed estimation $a^{spd}$:

(5) $$\begin{array}{cc} a^{dir} & \;\in \{\mathrm{move}\mathrm{straight},\;\mathrm{turn}\mathrm{left},\;\mathrm{turn}\mathrm{right}\}\\ a^{spd} & \;\in \{\mathrm{constant}\mathrm{speed},\;\mathrm{acceleration},\;\mathrm{deceleration},\;\mathrm{stop}\} \end{array}$$

resulting in 10 different meta-actions (*stop* is independent). Meta-action information can cover most driving scenarios and comprehensively simulate the vehicle’s behavior patterns in complex road environments.

The problem is defined as follows: by integrating multimodal perception information, including surrounding traffic participants’ observation information, ego vehicle motion information, and environmental visual information, DriveLLaVA based on VLM learns and simulates the behavior decision process of human drivers in real driving environments, planning the vehicle’s future high-level driving behavior. This method aims to improve the adaptability and safety of autonomous driving technology in complex traffic environments.

## 4. Dataset Generation

The purpose of dataset generation is to create an instruction-following dataset specifically for autonomous driving behavior decision tasks, based on real vehicle and surrounding traffic environment information. This dataset will be used for instruction fine-tuning of the VLM, enabling it to learn and simulate the behavior decision process in real human driving. We reviewed several existing autonomous driving datasets, including the KITTI, Argoverse, and Waymo Open Dataset:

(1) KITTI: An early benchmark for autonomous driving research, providing 3D point clouds and high-resolution images suitable for tasks such as object detection and SLAM. However, its dataset is relatively small, with limited and homogeneous scenes, lacking diversity and complex driving environments.
(2) Argoverse: Contains various sensor data and map information, suitable for perception and prediction tasks, but has a low sampling frequency and limited scene coverage.
(3) Waymo Open Dataset: A large-scale dataset containing various sensor data, such as 360-degree point clouds and high-resolution images, suitable for multiple autonomous driving tasks. However, the data are complex and voluminous, requiring high processing and storage capabilities.

This work selects nuScenes as the base dataset, an open dataset specifically designed for autonomous driving research. The dataset contains approximately 1000 real driving scenes collected from six cities, including Boston and Singapore, providing a rich and diverse set of urban driving environments. The nuScenes dataset features high-frequency sampling, with each scene lasting 20 s and sampled at a frequency of 2 Hz, resulting in a sequence of multiple keyframe camera images. Each keyframe image not only provides high-definition visual information but also comes with comprehensive sensor data. These sensor data detail ego vehicle motion data (like state data, trajectory data, and direction data) and surrounding traffic participants’ observation data (like category, current location, and predicted trajectory).

**Figure 1:**
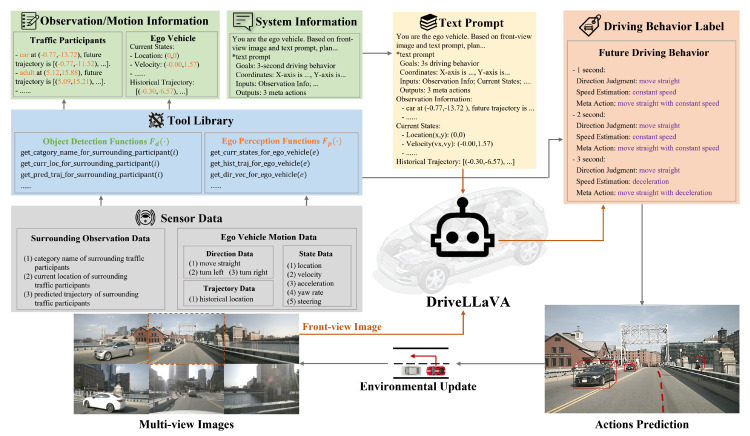
Instruction—following dataset generation process.

Given that the input for DriveLLaVA is multimodal prompt instructions and the output is fixed-format reasoning labels, this work constructs an instruction-following dataset specifically for autonomous driving behavior decision tasks based on the nuScenes dataset. This dataset contains a large number of image–text instructions paired with corresponding driving behavior labels. Figure 1 shows the construction process of the instruction-following dataset. We designed a tool library that includes a set of functions to extract DriveLLaVA input and output information from multi-view camera images and sensor data of each keyframe in the driving scenes.

To generate image–text instructions, first, object detection functions and ego perception functions in the tool library are used to extract specific sensor data. By processing these data, we obtained surrounding traffic participants’ observation information and ego vehicle motion information. Then, these data are encapsulated into a text prompt along with system information describing the background task. Finally, the front-view camera image of each keyframe in the driving scenes is selected and paired with the corresponding text prompt to generate image–text instructions, which are used to guide DriveLLaVA reasoning.

Similarly, to generate driving behavior labels, ego perception functions in the tool library are used to extract specific sensor data. By applying explicit rules to these data, we obtain the future driving behaviors that match each image–text instructions. These behaviors are then formatted into driving behavior labels and used to fine-tune the DriveLLaVA’s reasoning output.

### 4.1. Image–Text Instructions

To generate image–text instructions (I,T), all keyframe front-view camera images I from various driving scenarios in the nuScenes dataset are first selected, providing rich environmental visual information. Next, the sensor data ω accompanying each frame are processed to obtain surrounding traffic participants’ observation information O and ego vehicle motion information E. Specifically, this method uses object detection functions $F_{d}\left(\cdot \right)$ from the tool library to extract surrounding traffic participants’ observation data from the sensor data ω, such as category name $c^{i}$, current location $\left(x_{t_{obs}}^{i},y_{t_{obs}}^{i}\right)$, and predicted trajectory ${\left\{\left(x_{t_{obs}+k}^{i},y_{t_{obs}+k}^{i}\right)\right\}}_{k=1}^{T_{pred}^{\tau }}$. For each surrounding traffic participant, a fixed sentence is designed to describe these attributes, which together constitute the observation information O. Using ego perception functions $F_{p}\left(\cdot \right)$ from the tool library, this method extracts current state information of the ego vehicle from ω, including location $\left(x_{t_{obs}}^{e},y_{t_{obs}}^{e}\right)$, velocity $v_{t_{obs}}^{e}$, acceleration $a_{t_{obs}}^{e}$, yaw rate $w_{t_{obs}}^{e}$, and steering angle $\theta_{t_{obs}}^{e}$, as well as historical trajectory $h={\left\{\left(x_{t_{obs}-k}^{i},y_{t_{obs}-k}^{i}\right)\right\}}_{k=1}^{T_{hist}}$. These are embedded into the fixed text to generate ego vehicle motion information E. Finally, system information for background description, including task objective and coordinate, is provided, integrating it with surrounding traffic participants observation information O and ego vehicle motion information E to form text prompt T, as shown in the upper part of Figure 1. Each keyframe front-view camera image I is paired with the corresponding text prompt T, thus forming the image–text instruction (I,T).

### 4.2. Driving Behavior Labels

To enable the VLM to learn the outcomes of real human behavior decisions, this method generated driving behavior labels A that match the image–text instructions (I,T). The specific process is shown in Algorithm 1. The driving behavior label set A is initialized (line 1). Next, ego perception functions $F_{p}\left(\cdot \right)$ in the tool library are used to extract the adjacent speeds of the ego vehicle in the future $T_{pred}^{a}$ timesteps from the sensor data ω. By evaluating their numerical relationships, the longitudinal behavior label $a^{spd}\in A$ (speed estimation) is determined. Firstly, if both adjacent speeds are below the specified speed threshold ε, $a^{spd}$ is set to “*stop*”. Secondly, if the absolute difference between adjacent speeds is less than the speed threshold ε, $a^{spd}$ is set to “*constant speed*”. Finally, the size relationship between adjacent speeds is assessed: if $v_{t_{obs}+t}^{e}$ is greater than $v_{t_{obs}+t-1}^{e}$, $a^{spd}$ is set to “*acceleration*”; if $v_{t_{obs}+t}^{e}$ is less than $v_{t_{obs}+t-1}^{e}$, $a^{spd}$ is set to “*deceleration*” (lines 3–13). The ego vehicle’s direction command vector $V_{d}^{e}$ is also extracted using $F_{p}\left(\cdot \right)$ and based on its value type, the lateral behavior label $a^{dir}\in A$ (direction judgment) is determined. When $V_{d}^{e}$ is [1, 0, 0], $a^{dir}$ is set to “*turn right*”; when $V_{d}^{e}$ is [0, 1, 0], $a^{dir}$ is set to “*turn left*”; and when $V_{d}^{e}$ is [0, 0, 1], $a^{dir}$ is set to “*move straight*” (lines 14–21). After obtaining the longitudinal and lateral behavior labels, we combine them into meta-actions *a* (“*stop*” is independent) and add them to driving behavior set A (lines 22–24).
**Algorithm 1** Driving behavior labeling algorithm**Input:** image–text instructions (***I***, ***T***), sensor data ***ω***, speed threshold ε**Output:** driving behavior label set A      1: Initialize A=∅      2: **for** each element in (I,T) **do**      3:       **for** t←1 to $T_{\mathrm{pred}}^{a}$ **do**      4:            $\left\{v_{t_{\mathrm{obs}}+t-1}^{e},v_{t_{\mathrm{obs}}+t}^{e}\right\}=F_{p}\left(\omega \right)$      5:             **if** $v_{t_{\mathrm{obs}}+t-1}^{e}<\varepsilon$ and $v_{t_{\mathrm{obs}}+t}^{e}<\varepsilon$ **then**      6:                $a_{j}:=“\mathrm{stop}”$      7:             **else if** $\left|v_{t_{\mathrm{obs}}+t}^{e}-v_{t_{\mathrm{obs}}+t-1}^{e}\right|<\varepsilon$ **then**      8:                $a_{j}^{\mathrm{spd}}:=“\mathrm{constant speed}”$      9:             **else if** $v_{t_{\mathrm{obs}}+t}^{e}>v_{t_{\mathrm{obs}}+t-1}^{e}$ **then**      10:               $a_{j}^{\mathrm{spd}}:=“\mathrm{acceleration}”$      11:           **else**      12:               $a_{j}^{\mathrm{spd}}:=“\mathrm{deceleration}”$      13:           **end if**      14:           $V_{d}^{e}=F_{p}\left(\omega \right)$//$V_{d}^{e}$ is directional command vector      15:           **if** $V_{d}^{e}=\left[1,0,0\right]$ **then**      16:               $a_{j}^{\mathrm{dir}}:=“\mathrm{turn right}”$      17:           **else if** $V_{d}^{e}=\left[0,1,0\right]$ **then**      18:               $a_{j}^{\mathrm{dir}}:=“\mathrm{turn left}”$      19:           **else**      20:               $a_{j}^{\mathrm{dir}}:=“\mathrm{move straight}”$      21:           **end if**      22:           $a_{i}:=a_{j}^{\mathrm{dir}}\times a_{j}^{\mathrm{spd}}$      23:           Δ Append meta action $a_{j}$ to A      24:       **end for**      25: **end for**

**Figure 2:**
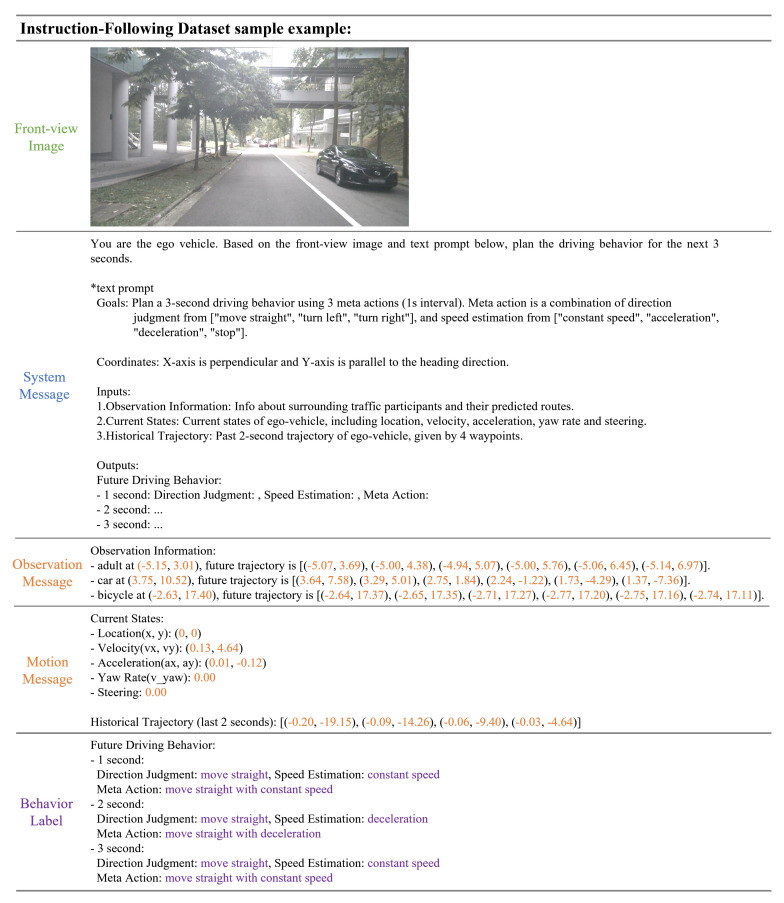
Example of instruction−following dataset sample, including Front-view Image, System Message, Observation Message, Motion Message, and Behavior Label.

Based on the above steps, this work constructs an instruction-following dataset containing approximately 24K image–text instructions and corresponding driving behavior label samples. ChatGPT-4 was used to refine these samples into more concise forms. Compared to other datasets, the instruction-following dataset constructed in this work has the following advantages. (a) Diverse and Complex Scenarios: It includes 24K driving images collected from a wide variety of urban driving environments. This diversity ensures the dataset covers numerous complex driving conditions, enhancing the robustness of the model’s decision capabilities. (b) Multimodal Input Prompts: It integrates high-resolution visual information with comprehensive sensor data, like multi-view camera images and text prompts. This multimodal information provides a richer context for training, allowing the model to make more informed and accurate driving decisions. (c) Rich image–text Instructions and Precise Driving Behavior Labels: The rich image–text instructions generated from front-view camera images and sensor data, along with the precise driving behavior labels created through the designed tool library, provide the model with a large number of training samples and help in better learning and simulating human driving behavior decision processes. An example of a sample from the instruction-following dataset is shown in Figure 2.

## 5. Methodology

### 5.1. Model Architecture

**Figure 3:**
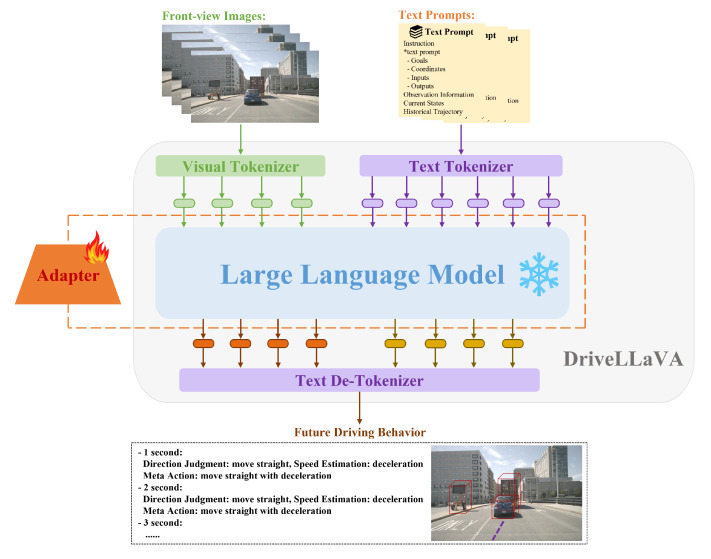
Overall architecture of DriveLLaVA divided into three parts: Multimodal data encoding and alignment, lightweight fine-tuning of the LLM with adapter, and fixed-format decoding of future driving behavior.

The DriveLLaVA model architecture is divided into three main parts, as shown in Figure 3: multimodal data encoding and alignment, lightweight fine-tuning with an adapter for the LLM base, and fixed-format decoding of future driving behavior.

Firstly, the multimodal data-encoding and alignment part is mainly responsible for converting visual images and text prompts into a format that the LLM can process. The text prompts are encoded through a text tokenizer, while the visual images are encoded through a visual tokenizer and aligned with the text embedding space. These two different modalities of data are converted into token sequences with unified embedding space dimensions and are input together into the LLM for processing.

Secondly, to ensure that the DriveLLaVA model can accurately adapt to the current task, adapter technology is used for lightweight fine-tuning of the LLM. This adapter fine-tuning approach enhances the LLM’s ability to understand and process the encoded and aligned multimodal data, allowing it to generate effective prediction tokens. Additionally, by optimizing the number of LLM parameters, this approach significantly reduces training costs while ensuring that the model maintains high performance and efficiency.

Finally, the predicted tokens generated by the LLM are decoded using a text de-tokenizer. In the decoding process, to improve the efficiency of data extraction, the predicted future driving behavior is designed in a fixed format: a text-based set of meta-actions, with each meta-action subdivided into a combination of direction and speed components.

#### 5.1.1. Multimodal Data Encoding and Alignment

The input of DriveLLaVA is the multimodal information for each driving scene frame, namely the image–text instruction (I,T). First, the text prompt T is converted into a series of text tokens $Y_{T}$ using a text tokenizer f(·):

(6) $$Y_{T}=f\left(T\right)$$

Secondly, the front-view camera image I is processed using a visual tokenizer. The visual tokenizer comprises an image encoder g(·) and an image-language connector δ. Following the work of [40], we adopt CLIP-ViT-L/336px as the image encoder, which has been pretrained on a large number of image–text pairs to extract the visual features of the input image $V_{I}$:

(7) $$V_{I}=g\left(I\right),\;\mathrm{where}\;V_{I}=\left[V_{I}^{\left[CLS\right]},V_{I}^{1},V_{I}^{2},\cdots ,V_{I}^{256}\right]$$

where the first token of the visual features, $V_{I}^{\left[CLS\right]}$, represents the global feature of the image, while the other 256 tokens, $V_{I}^{k},k=1,2,\ldots ,256$, correspond to the local features of various patches in the image. The global feature and all local features are concatenated to obtain the complete visual features of the input image ${\hat{V}}_{I}$:

(8) $${\hat{V}}_{I}=V_{I}^{\left[CLS\right]}⊕V_{I}^{1}⊕\cdots ⊕V_{I}^{256}$$

where ⊕ denotes concatenation. We use a two-layer perceptron (MLP) as the image–language connector to map the complete visual features ${\hat{V}}_{I}$ into the text embedding space:

(9) $$Y_{I}=\delta \cdot {\hat{V}}_{I}$$

where $Y_{I}$ represents the image tokens, which have the same embedding space dimensions as the text tokens $Y_{T}$.

Finally, all the obtained tokens are concatenated, where $Y_{L}=Connect\left(Y_{I},Y_{T}\right)$, and processed as the input to the LLM.

#### 5.1.2. Lightweight Fine-Tuning of the LLM with the Adapter

This work uses LoRA as an adapter for lightweight fine-tuning of the LLM. The core idea of LoRA fine-tuning approach is to achieve dynamic adjustment of model weights by adding low-rank matrices to the weight matrix of the pretrained model. This fine-tuning approach allows the effective adjustment of the model to a specific task by introducing only a small number of parameters while keeping the weight parameters of the pretrained model unchanged.

**Figure 4:**
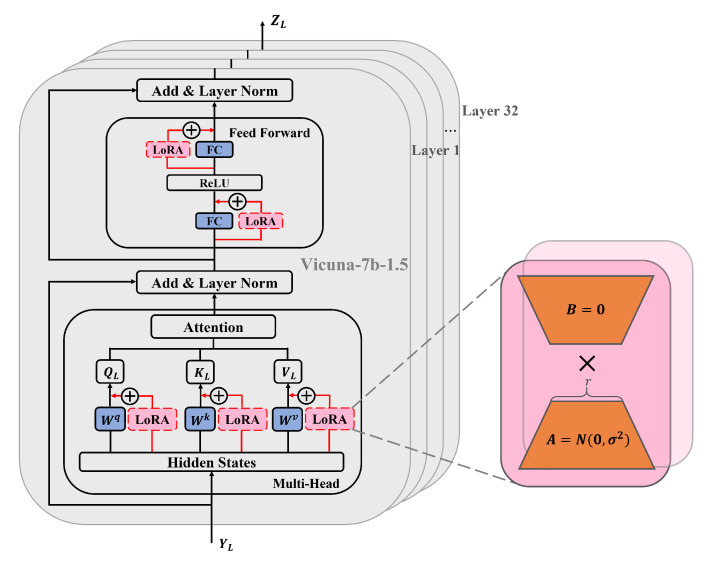
Lightweight fine-tuning of the LLM with the adapter.

As shown in Figure 4, Vicuna-7b-1.5 is used as the LLM, which consists of 32 layers of transformers and has excellent instruction-following capabilities for language tasks. In this work, LoRA is added to the linear layers of each transformer in Vicuna-7b-1.5, including the Multi-Head Attention (MHA) and Feed-Forward Network (FFN) layers. First, the LLM input $Y_{L}$ enters the Multi-Head Attention layer. In this layer, LoRA is added to each attention matrix, including query matrix $Q_{L}$, key matrix $K_{L}$, and value matrix $V_{L}$. LoRA is a bypass module that includes a down-projection matrix and an up-projection matrix. During training, the input and output dimensions of the Multi-Head Attention layer remain unchanged, and the linear transformation matrices of each attention matrix are frozen. Only the added down-projection matrix A and up-projection matrix B are trained, with A initialized using a random Gaussian distribution and B initialized using a zero matrix. Therefore, the calculation process of the entire Multi-Head Attention mechanism can be represented as follows:

(10) $$MultiHead\left(Q_{L},K_{L},V_{L}\right)=Concat\left(head_{1},\cdots ,head_{h}\right)W^{o}$$

(11) $$\begin{array}{cc} head_{h}= & Attn\left(Q_{L},K_{L},V_{L}\right)=softmax\left(\frac{Q_{L}K_{L}^{T}}{\sqrt{d_{k}}}\right)V_{L}\\ \; & where\;\left\{\begin{array}{c} Q_{L}=\left(W_{h}^{q}+B_{h}^{q}A_{h}^{q}\right)Y_{L}\\ K_{L}=\left(W_{h}^{k}+B_{h}^{k}A_{h}^{k}\right)Y_{L}\\ V_{L}=\left(W_{h}^{v}+B_{h}^{v}A_{h}^{v}\right)Y_{L} \end{array}\right. \end{array}$$

where $W_{h}^{q}\in R^{d_{model}\times d_{k}}$, $W_{h}^{k}\in R^{d_{model}\times d_{k}}$ and $W_{h}^{v}\in R^{d_{model}\times d_{v}}$ are the linear transformation matrices of query matrix $Q_{L}$, key matrix $K_{L}$ and value matrix $V_{L}$ for each attention head h. $A_{h}^{q}\in R^{d_{model}\times r}$, $A_{h}^{k}\in R^{d_{model}\times r}$, and $A_{h}^{v}\in R^{d_{model}\times r}$ are the corresponding down-projection matrices. $B_{h}^{q}\in R^{r\times d_{k}}$, $B_{h}^{k}\in R^{r\times d_{k}}$ and $B_{h}^{v}\in R^{r\times d_{v}}$ are the corresponding up-projection matrices, with $r\ll min(d_{model},d_{k/v})$. $W^{o}\in R^{hd_{v}\times d_{model}}$ represents the output transformation matrix.

Furthermore, by performing a residual connection and layer normalization, we obtain $H_{L}$:

(12) $$H_{L}=LayerNorm\left(Y_{L}+MultiHead\left(Q_{L},K_{L},V_{L}\right)\right)$$

$H_{L}$ then goes through the feed-forward network layer rFFN(·), which consists of two fully connected layers and a ReLU activation function. We add LoRA to both fully connected layers, and the training method is the same as mentioned above. The weight matrices of each fully connected layer are frozen, and only the down-projection matrix A and up-projection matrix B are trained. Then, by performing another residual connection and layer normalization, we obtain the output of a single transformer layer $S_{L}$:

(13) $$rFFN\left(H_{L}\right)=ReLU\left(\left(W_{1}^{f}+B_{1}^{f}A_{1}^{f}\right)H_{L}\right)\left(W_{2}^{f}+B_{2}^{f}A_{2}^{f}\right)$$

(14) $$S_{L}=LayerNorm\left(H_{L}+rFFN\left(H_{L}\right)\right)$$

where $W_{1}^{f}\in R^{d_{model}\times d_{ff}}$ is the weight matrix of the first fully connected layer and $W_{2}^{f}\in R^{d_{ff}\times d_{model}}$ is the weight matrix of the second fully connected layer. $A_{1}^{f}\in R^{d_{model}\times r}$ and $A_{2}^{f}\in R^{d_{ff}\times r}$ are the corresponding down-projection matrices. $B_{1}^{f}\in R^{r\times d_{ff}}$ and $B_{2}^{f}\in R^{r\times d_{model}}$ are the corresponding up-projection matrices, with $r\ll min(d_{model},d_{ff})$.

Finally, $S_{L}$ goes through L layers of transformers, repeating the above process, to obtain the final prediction tokens $Z_{L}$ of the LLM, which contain all the output information for the current task.

#### 5.1.3. Fixed-Format Decoding of Future Driving Behavior

To facilitate extraction and subsequent processing, predicted future driving behavior A is decoded into a fixed format. The specific decoding process is as follows:

After obtaining the prediction tokens $Z_{L}$ generated by the LLM, the text de-tokenizer Φ is used to decode them back into human language. During the decoding process, future driving behavior A is designed as text-based meta-action set, with each meta-action subdivided into a combination of direction and speed components. This fixed-format decoding of driving behavior enhances the transparency and interpretability of the model throughout the decision process. The above decoding process can be represented as

(15) $$A=\Phi (Z_{L}),\;where\;A\in T$$

where A∈T indicates that future driving behavior belongs to the text domain.

### 5.2. Model Fine-Tuning

To enable DriveLLaVA to understand and handle the current task, we perform instruction fine-tuning using the instruction-following dataset constructed in Section 4. Considering that the output of DriveLLaVA is only a text corpus, we use the cross-entropy loss $L\left(\theta \right)$ to supervise the model’s output. The cross-entropy loss is defined as follows:

(16) $$L\left(\theta \right)=-\left(logp\left(A|I,T\right)\right)=-\left(\sum_{j=1}^{M}logp_{\theta }\left(a_{j}|I,T,A_{<j}\right)\right)$$

where θ represents the trainable parameters and $A_{<j}$ represents all the driving behavior sequences before the current predicted meta-action $a_{j}$.

Throughout the fine-tuning process, to significantly reduce training costs, we adopt the LoRA approach to efficiently optimize the number of model parameters. This means keeping the weights of the image encoder and the LLM frozen and updating only the weights of the image-language connector and LoRA by minimizing the cross-entropy loss. This allows DriveLLaVA to accurately infer future behavior strategies consistent with actual human driving decisions.

## 6. Experiment

This section evaluates the capabilities of DriveLLaVA through extensive experiments on an instruction-following dataset. First, the experimental setup and evaluation metrics are introduced. Next, this method is compared with other baseline methods, demonstrating its superior performance in behavior decisions. An ablation study is then conducted to verify the compatibility and optimization design of our method. Additionally, a comparative experiment between in-context learning and instruction fine-tuning is designed, proving the necessity of model fine-tuning. Finally, extensive qualitative analysis of behavior decision examples in various complex driving scenarios demonstrates DriveLLaVA’s robustness and generalization ability.

### 6.1. Experimental Setup

The entire instruction-following dataset is divided into a training set and a validation set. The training set is used to instruction fine-tune the model, and the model’s performance is evaluated on the validation set, which ensures a fair comparison with baseline methods. During the instruction fine-tuning phase, the AdamW optimizer is used to update the loss function, with the initial learning rate set to 2 × 10^−5^ and decayed to 0 using cosine annealing. The input image size is 336 × 336. Considering the large number of parameters in the entire model, the LoRA fine-tuning approach is used to optimize the number of model parameters and save training costs. The dimension of the down-projection matrix *r* is set to 128, and the target modules are set to all linear layers of the LLM backbone in the model. The model is trained for five epochs, with a batch size of three per GPU. The entire training process used 8 RTX 4090 (24 GB) GPUs.

To quantitatively evaluate the effectiveness of our method in behavior decision tasks, meta-action accuracy is used as the evaluation metric and further divides the overall accuracy into direction and speed components. By comparing the model’s inference results with the manually collected ground truth labels, we comprehensively reflect the accuracy of the decisions and their similarity to human driving behavior. Following common practice, our method evaluates the behavior decision results within a 3-s time horizon.

### 6.2. Dataset Analysis

Table 1 presents a comparison with the previous datasets designed for driving understanding with natural language. The instruction-following dataset constructed in this work can simultaneously include perception, reasoning, decision, and alignment, offering a more reasonable and comprehensive reflection of the behavior decision process in actual human driving. Moreover, the vast amount of image–text instructions and corresponding driving behavior labels generated from the rich camera images and sensor information in the nuScenes dataset also enhances the interpretability and transparency of the entire decision process.

### 6.3. Comparison with Baseline Methods

To demonstrate DriveLLaVA’s superior performance in behavior decision, this work compares it with advanced baseline methods, which encompass the rule-based method, the LM-based method, and the end-to-end IL method. They have shown satisfactory results in behavior decision tasks, providing a robust benchmark for comparing the performance of DriveLLaVA. These baseline methods include

(1) **InstructBLIP** [51] and **Apollo** [52]: Behavior decision methods based on VLM and Finite State Machines (FSMs), achieving few-shot adaptation by providing input/decision pairs.
(2) **DriveVLM** [44]: An autonomous driving system that enhances scene understanding and planning capabilities through VLM, integrating a unique combination of chain-of-thought (CoT) modules to achieve scene description, scene analysis, and hierarchical planning.
(3) **DriveMLM** [53]: An autonomous driving behavior planning module built on LLM, using driving rules, user commands, and sensor data as inputs to make driving decisions and provide explanations.
(4) **DriveLM-Agent** and **DriveLM-Agent(GT)** [45]: A decision module based on VLM, trained on internet-scale data, making decisions through Visual Question Answering (VQA) adaptation.
(5) **TransFuser++** [54]: An end-to-end autonomous driving system based on the IL method, tested on CARLA, with excellent route-following capabilities.

Table 2 shows a comparison of DriveLLaVA with baseline methods in terms of behavior decision performance. DriveLLaVA significantly outperformed baseline methods in meta-action accuracy metrics, demonstrating the accuracy of this approach in generating human-like driving behaviors. Specifically, DriveLLaVA achieves the highest meta-action accuracy at 78.97%, an 8.78% improvement over the second-best method, TranFuser++. Its accuracy levels in the direction and speed components are 90.99% and 82.42%, respectively, surpassing baseline methods and showcasing its decision capabilities across various directional and speed decisions. Moreover, in driving scenarios such as straight roads and intersections, DriveLLaVA consistently exhibits superior behavior decision ability, indicating its high efficiency and practicality in common driving scenarios. Compared to non-large model (LM)-based methods, such as Apollo and TransFuser++, DriveLLaVA leverages the general knowledge and understanding capabilities of VLM to plan behavior strategies that more closely resemble those of human drivers, especially in extreme driving scenarios, such as adverse weather, unexpected pedestrians, or obstacles, where meta-action accuracy is significantly improved.

Compared to LM-based methods, such as DriveVLM, DriveMLM and DriveLM-Agent, DriveLLaVA also has performance advantages. These methods heavily rely on intensive multi-turn QA pairs (QAs) and chain-of-thought reasoning, making their systems complex and time-consuming. In contrast, DriveLLaVA only uses the multi-modal perception information from the ego vehicle as input observations, relying on the powerful prior knowledge capability of VLM for behavior decisions, making it much simpler than these methods. Thus, DriveLLaVA’s exceptional meta-action accuracy underscores its prowess in behavior decision, substantially enhancing autonomous driving safety. The integration of multi-turn QAs holds potential for further performance enhancements.

### 6.4. Ablation Study

An ablation study was conducted to verify the compatibility and optimization design of this method. The ego vehicle’s driving behavior is represented as a set of meta-actions over multiple future timesteps. A series of ablation experiments were designed by varying the granularity of meta-actions, i.e., the number of meta-actions representing future driving behavior, to observe the performance differences of DriveLLaVA. Specifically, the ego vehicle’s future 3-s driving behavior was represented with 1 meta-action (3 s interval), 3 meta-actions (1 s interval), and 6 meta-actions (0.5 s interval), using meta-action accuracy as the evaluation metric. The results of the ablation experiment are provided in Table 3. It was observed that the model performs optimally when the meta-action granularity is set to 3. However, the model’s performance decreases when the granularity is set to 1 or 6. Nevertheless, these configurations generally perform similarly to baseline methods. Thus, the ablation study on meta-action granularity preliminarily verifies the compatibility and optimization design of this method.

### 6.5. Instruction Fine-Tuning vs. In-Context Learning

In-Context Learning and instruction fine-tuning are two popular strategies for guiding VLM to perform specific tasks. Although the instruction fine-tuning strategy in this work performs excellently in behavior decision tasks, it raises the question of whether In-Context Learning could achieve comparable results. To this end, an In-Context Learning experiment was designed, where the prompts and expected outputs from the training set were used as new exemplar inputs to guide DriveLLaVA. The results were then compared with instruction fine-tuning.

As shown in Table 4, instruction fine-tuning significantly outperforms In-Context Learning in terms of meta-action accuracy. This is mainly because, in In-Context Learning, the model’s contextual observation window is very limited. In this work, due to the inherent sequence length limitation of DriveLLaVA, it can only accommodate up to two examples at a time. Therefore, in behavior decision, instruction fine-tuning performs noticeably better than In-Context Learning, demonstrating that the instruction fine-tuning strategy is indispensable in this work.

### 6.6. Visualization and Qualitative Analysis

**Figure 5:**
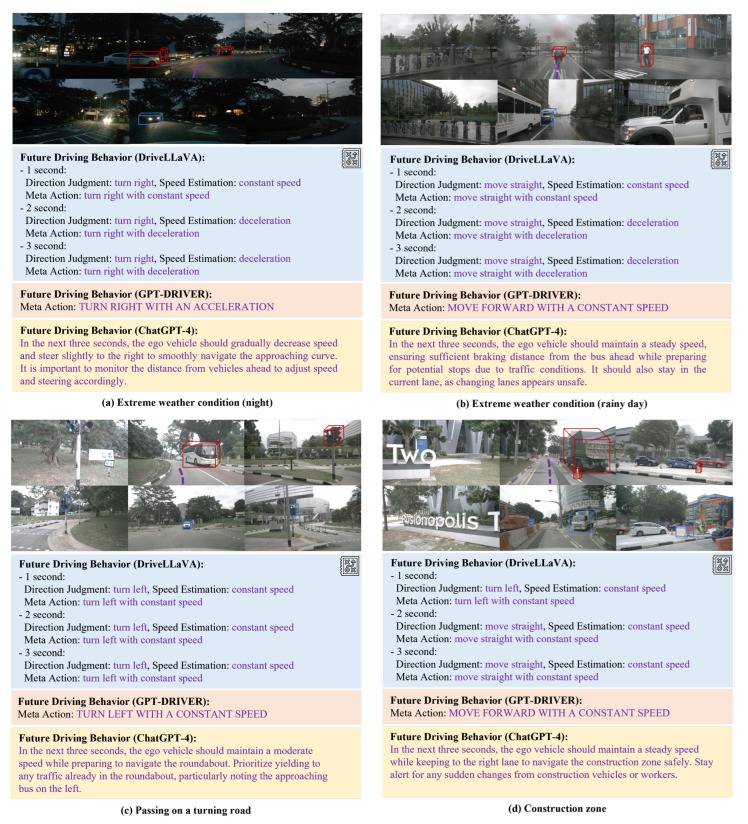

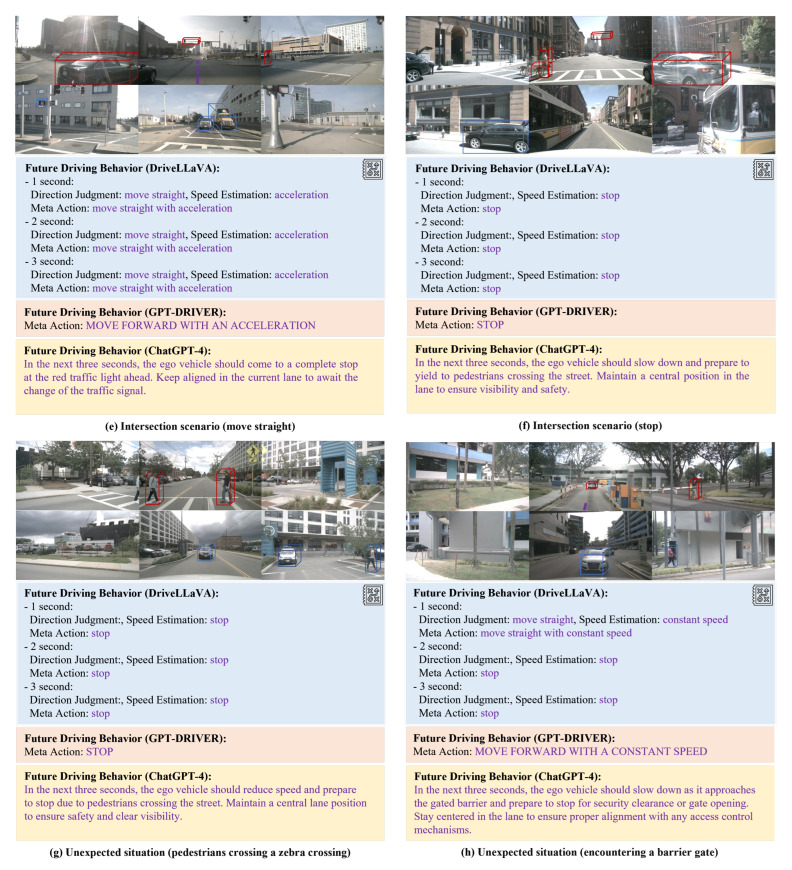
Visualization examples of behavior decisions.

**Figure 6:**
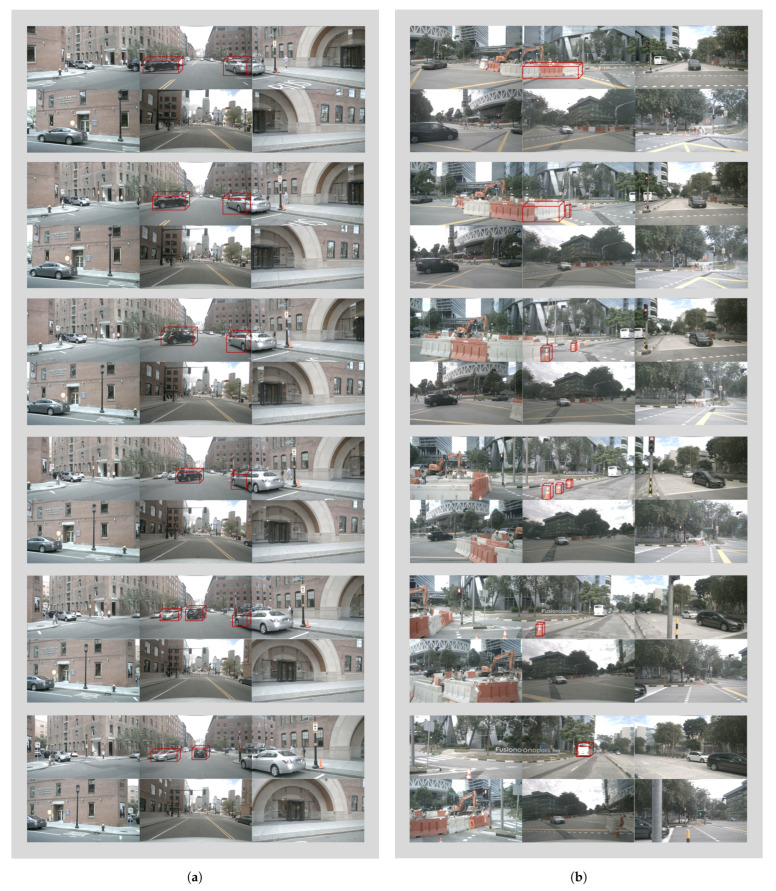
Driving video of DriveLLaVA. Video (**a**) shows the driving behavior of the ego vehicle when encountering lane change and passing by other vehicles; Video (**b**) shows the lane change behavior of the ego vehicle in a construction zone.

To demonstrate the robustness and generalization ability of DriveLLaVA, Figure 5 provides a visual comparison of DriveLLaVA, GPT-DRIVER, and ChatGPT-4 on the task of behavior decision. These scenarios include extreme weather conditions (such as night and rainy days), construction zones, intersections, and unexpected situations during driving (such as pedestrians crossing a zebra crossing or encountering a barrier gate). As shown in the figure, DriveLLaVA accurately identifies key traffic participants and predicts their future actions in various complex driving scenarios. Based on these observations and predictions, it generates comprehensive and precise high-level driving behaviors, using a meta-action sequence to depict behavioral states every second. In contrast, GPT-DRIVER uses a single meta-action to describe the entire future 3-s driving behavior, which is less comprehensive and more error-prone. Meanwhile, the driving behavior descriptions by ChatGPT-4 are overly verbose and lack intuitiveness. Thus, this approach provides greater comprehensiveness and intuitiveness and is comparable to the current best, ChatGPT-4. For example, in Figure 5a, DriveLLaVA can promptly detect the black car ahead and the white car in the left lane about to merge into the current lane during a low-visibility nighttime roundabout driving scenario. It then generates a right turn and deceleration action to avoid collisions with these cars. As shown Figure 5d, while driving through a construction zone, DriveLLaVA can recognize all obstacles in the road (such as traffic cones) and construction trucks. It then generates a straight-ahead action, maintaining a steady speed to pass through the construction zone. As shown in Figure 5g, DriveLLaVA can promptly spot a pedestrian crossing the zebra crossing ahead and appropriately generate a stop action to yield to the pedestrian. Figure 6 shows the complete driving process inferred by DriveLLaVA for the next three seconds. In Figure 6a, the future driving behavior of the ego vehicle is depicted when it encounters a sudden lane change from the left lane into the straight lane, and then other vehicles pass each other. Figure 6b illustrates the ego vehicle’s left lane change behavior in a construction zone.

Therefore, by quantitatively analyzing the results of DriveLLaVA in multiple experiments, the effectiveness and superior decision capabilities of this method are demonstrated. Through visualization and qualitative analysis of behavior decision examples of DriveLLaVA in various complex traffic driving scenarios, the robustness and generalization ability of DriveLLaVA are further illustrated.

## 7. Conclusions

This paper introduces DriveLLaVA, a novel VLM dedicated to autonomous driving behavior decision tasks. A novel instruction-following dataset is developed, containing a large number of interpretable image–text instructions and driving behavior labels, used for fine-tuning DriveLLaVA. During the fine-tuning process, the LoRA approach is employed to optimize model parameters and reduce training costs. DriveLLaVA can utilize multimodal perception information, including surrounding traffic participants’ observation information, ego vehicle motion information, and environmental visual information, to infer future behavior strategies consistent with human driving.

Through extensive experiments on the instruction-following dataset, DriveLLaVA demonstrated excellent performance, robustness, and generalization ability in behavior decisions, surpassing baseline methods. The implementation of DriveLLaVA offers research potential for the field of autonomous driving decisions. However, there are still some drawbacks and limitations. On the one hand, due to dataset limitations, our method only fine-tunes DriveLLaVA for the autonomous driving behavior decision task without a pretraining process for the model. Future work should first pretrain it using a larger-scale autonomous driving domain dataset to equip it with prior general knowledge about autonomous driving, followed by instruction fine-tuning for specific tasks. On the other hand, due to parameter limitations, VLM-based methods usually exhibit longer inference times compared to existing MLP-based methods, making it challenging to meet the real-time requirements of commercial driving applications. Future work should focus on distilling a smaller VLM or using a large VLM to guide a smaller VLM to optimize inference time.

## Figures and Tables

**Table 1:** Comparison of autonomous driving datasets for driving understanding.

Dataset	Perception	Reasoning	Decision	Alignment
Nuprompt [46]	**✓**			
NuScenes-QA [47]	**✓**	**✓**		
Rank2Tell [48]	**✓**	**✓**		
BDD-X [49]		**✓**	**✓**	
DRAMA [50]		**✓**	**✓**	
Ours	**✓**	**✓**	**✓**	**✓**

**Table 2:** Behavior decision performance.

Method	Type	Acc. (%) ↑	Meta Action	
			**Direction (%) ↑**	**Speed (%) ↑**
InstructBLIP	VLM	17.92	\	\
Apollo	FSM	18.53	\	\
DriveVLM	VLM	37.00	\	\
DriveMLM	LLM	39.50	\	\
DriveLM-Agent(GT)	VLM	60.25	65.22	80.12
DriveLM-Agent	VLM	61.45	84.73	72.20
TransFuser++	IL	70.19	90.68	73.29
**DriveLLaVA**	**VLM**	**18.97**	**90.99**	**82.42**

**Table 3:** Quantitative results of ablation study.

Method	Granularity	Acc. (%)	Meta Action	
			**Direction (%)**	**Speed (%)**
DriveLLaVA	1	59.45	88.04	63.94
	**3**	**78.97**	**90.99**	**82.42**
	6	73.24	90.23	79.78

**Table 4:** Instruction fine-tuning vs. In-Context Learning.

Method	Acc. (%)	Meta Action	
		**Direction (%)**	**Speed (%)**
DriveLLaVA (In-Context Learning)	38.92	75.28	43.37
DriveLLaVA (instruction fine-tuning)	**78.97**	**90.99**	**82.42**

## Data Availability

The data presented in this study are available upon request from the corresponding author.
